# Prevalence of *Borreliaceae* Spirochetes in Ticks Removed from Humans in Poland During 2018–2022

**DOI:** 10.3390/pathogens14121234

**Published:** 2025-12-03

**Authors:** Beata Wodecka, Valentyna Kolomiiets

**Affiliations:** 1 Department of Genetics and Genomics, Biology Institute, University of Szczecin, Felczaka 3c, 71-412 Szczecin, Poland; valentyna.kolomiiets@usz.edu.pl; 2 Doctoral School, University of Szczecin, Adama Mickiewicza 16, 70-384 Szczecin, Poland

**Keywords:** ticks, tick-borne diseases, *Borreliella*, *Borrelia miyamotoi*

## Abstract

Monitoring the occurrence of *Borreliaceae* spirochetes in ticks may provide an indication of the risks of acquiring Lyme borreliosis (LB) and Borrelia miyamotoi disease (BMD). All ticks obtained in our study from humans in the years 2018–2022 (n = 1232) were identified morphologically for species, sex and developmental stage. The detection of *Borreliaceae* spirochetes and species identification were performed by nested PCR based on the *flaB* gene fragment and the region between the *mag* and *trnI* genes. Two species of ticks were identified: *Ixodes ricinus* (96.9%) and *Dermacentor reticulatus* (3.1%). The infection of *I. ricinus* ticks with *Borreliaceae* spirochetes was found to reach 18.3%, including *B. miyamotoi* (2.5%). Among *Borreliella* species, *Bl. afzelii* was the most frequent, followed by *Bl. burgdorferi*, *Bl. spielmanii*, *Bl. valaisiana*, *Bl. garinii*, *Bl. bissettiae*, *Bl. californiensis* and *Bl. carolinensis*. *Borreliaceae* spirochetes were also found in *D. reticulatus* ticks, of which *Bl. afzelii* and *B. miyamotoi* were the most common. In conclusion, ticks affecting humans in Poland represent a real risk of infection with *Borreliaceae* spirochetes, and knowledge of the prevalence and distribution of these bacteria is an important tool in assessing the risks of LB and BMD.

## 1. Introduction

Lyme borreliosis (LB) is the most prevalent tick-borne disease in Europe, with more than 128,000 inhabitants affected annually [1]. In Poland, the incidence of the disease is increasing annually, at least doubling with each decade, from 9.36 cases per 100,000 inhabitants in 2003 through 33.2/100,000 in 2013 to 67.1 per 100,000 inhabitants in 2023 [2]. However, the assessment of the actual incidence of LB is hampered by diagnostic problems and limitations in the reporting of cases in different European countries [1]. The etiological agents of LB are bacterial species formerly referred to as *Borrelia burgdorferi* sensu lato complex and currently classified as *Borreliella* [3,4], although two hypotheses still exist, according to which this taxon should have the rank of genus or subgenus within the genus *Borrelia* [5,6]. At least five of the 26 species of the genus *Borreliella* are responsible for the LB incidence in Europe, namely *Bl. burgdorferi*, *Bl. afzelii*, *Bl. garinii*, *Bl. spielmanii* and *Bl. bavariensis* [7,8,9]. These species cause distinct forms of LB, especially the first three mentioned, of which *Bl. burgdorferi* is associated with rheumatoid symptoms, *Bl. afzelii* causes skin infections, and *Bl. garinii* as well as *Bl. bavariensis* have an affinity for the nervous system [8,10]. In addition, three other species present in Europe have lower pathogenic potential, namely *Bl. lusitaniae*, *Bl. valaisiana* and *Bl. bissettiae* [11]. On the other hand, a study from North America, involving the verification of a more precise serological test for the differentiation of species of the *Borreliaceae* family in people with symptoms of LB, showed the presence of *Bl. californiensis* [11], which has also recently been detected in the European continent [9,12]. Such a multiplicity of actual as well as potentially pathogenic species further complicates the diagnosis of LB as the distribution of individual spirochete species varies geographically, influenced by the availability of their distinct animal reservoirs [13,14,15]. Recently, the pathogenic impact on humans in Europe of *Borrelia miyamotoi*, causing a type of hard tick-borne relapsing fever called Borrelia miyamotoi disease (BMD), has also been identified [16,17], but there are few data on the incidence in different areas of Europe [18,19].

All *Borreliella* species found in Europe, with the exception of *Bl. turdi*, are spread by the same tick species, namely *Ixodes ricinus*, similarly to *B. miyamotoi* [9,20,21,22]. This tick species is the most widespread in Europe and is associated with deciduous and mixed forest environments, while its occurrence in the last few decades has been clearly expanding towards the north of the continent and into areas higher above sea level [23]. *Ixodes ricinus* significantly predominates among tick species attacking humans, and the nymph stage is the main contributor to these attacks [24]. The increase in the number of ticks observed with each decade, including in urban areas, and especially the prolonged season of activity throughout the year of these arachnids, has its sources in environmental changes, which mainly happen due to human activity in terms of agricultural land use and forest management, resulting in changes in the activity of various animal species and climate change [25]. This, in turn, is reflected in the increasing risk of tick attacks against humans and thus the risk of acquiring tick-borne diseases, one of the most common and dangerous of which is Lyme borreliosis [1].

Due to the existence of a common vector for the different species of the genus *Borreliella* and *B. miyamotoi*, knowledge of their spread in ticks may be crucial in understanding the epidemiology of both LB and BMD, as well as for the prevention and diagnosis of these diseases. Data on the spread of individual species of these bacteria in ticks collected from humans in Europe are limited, and the identification of spirochete species carried out is not precise [26,27,28,29,30,31,32,33,34,35]. In Poland, more precise data on the distribution of individual species of *Borreliaceae* spirochetes in ticks collected from humans are scarce [36], with most concerning ticks collected from vegetation [9,37,38,39] or animals [12,40,41,42,43,44,45]. It is therefore worth extending these analyses, as data determined from studies of ticks collected from humans may provide specific information about the risk of human exposure to tick-borne diseases, especially those caused by *Borreliaceae* spirochetes. The aim of our study was to assess the spread and prevalence of LB- and BMD-causing bacteria in ticks removed from humans in Poland over five consecutive years, taking into account the tick’s feeding status and its potential impact on infection with individual species of the *Borreliaceae* family.

## 2. Materials and Methods

**Collection of ticks and their identification.** The study presented here was conducted for five consecutive years, i.e., from 2018 to 2022, on ticks found attached to human skin. Ticks were collected throughout Poland from January to November in the years mentioned and then delivered by patients to the Department of Genetics and Genomics at the University of Szczecin in sealed, ethanol-filled containers, up to seven days after removal from the skin by a physician or the patient themselves. Ticks were identified morphologically for species and also for stage and sex determination according to a standard taxonomic key [46].

**Contamination control procedures.** In order to avoid contamination of the tested biological material, further steps of the research—i.e., DNA isolation and preparation of the PCR reaction mixture and positive samples for sequencing—were carried out in separate, hermetically locked rooms.

**DNA isolation.** Individual tick specimens (larvae, nymphs and adults) were placed in separate Eppendorf tubes, in which a steel bead was placed, and the entirety was suspended in 100 uL of PBS buffer (Eurx, Gdansk, Poland). Such prepared samples were homogenized at 50 Hz for 5 min using a TissueLyser LT homogenizer (Qiagen, Hilden, Germany). Homogenized samples were subjected to DNA isolation using the GeneMatrix Tissue and Bacterial DNA Purification Kit (Eurx, Gdansk, Poland), according to the manufacturer’s protocol.

**Detection of *Borreliaceae* spirochete DNA and species identification.** A sensitive and highly specific nested PCR method was used to detect spirochete DNA from the *Borreliaceae* family. Two sets of primers specific to the entire *Borreliaceae* family were used, allowing the amplification of DNA fragments for two molecular markers: the *flaB* gene and the intergenic region flanked by the *mag* and *trnI* genes (Table 1). The use of two molecular markers based on DNA sequences located in different areas of the genome was employed to confirm the detection of specific taxa.

Species identification was carried out using two different methods, enabling the independent confirmation of the correctness of species identification: (1) the sequence length polymorphism (SLP) occurring within the DNA sequence obtained with primers mag-435F and trni-65R [9,45]; (2) the restriction fragment length polymorphism of the *flaB* gene fragment obtained with primers 220f and 823r using the enzyme DdeI (Thermo Fisher Scientific, Waltham, MA, USA) and, if required, one of PsuI, SatI or VspI (Thermo Fisher Scientific) in the next step, as previously described [9,47].

In each PCR run, DNA from the *Bl. afzelii* reference strain VS461 (German Collection of Microorganisms and Cell Cultures—DSMZ, Leibniz, Germany) was the positive control and TE buffer was the negative control. The electrophoretic separation of PCR products was performed on a 1.5% agarose gel (Bioshop, Boston, MA, USA), and visualization and documentation were performed as previously described [48].

**DNA sequencing of *Borreliaceae* spirochetes and sequence similarity analysis.** In order to confirm the accuracy of the methods for identifying species of *Borreliaceae* spirochetes established on the basis of polymorphisms in the length of the *mag-trnI* intergenic spacer sequence and polymorphisms in the lengths of restriction fragments of the *flaB* gene, both markers were sequenced. Sequencing of *flaB* gene fragments with internal primers FL120F/FL908R and DNA fragments with internal primers mag-435F and trnI-65R was performed for positive samples representative of different species of the *Borreliaceae* family. DNA sequencing was performed at Macrogen Europe (Amsterdam, The Netherlands).

For both the *flaB* gene and the *mag-trnI* intergenic spacer, 35 sequences each were deposited in the GenBank database. The *flaB* gene sequences were assigned the following accession numbers: PV683492-PV683501 (*Bl. afzelii*), PV683502-PV683504 (*Bl. garinii*), PV683505-PV683508 (*Bl. burgdorferi*), PV683509-PV683511 (*Bl. valaisiana*), PV683512-PV683515 (*Bl. spielmanii*), PV683516-PV683518 (*Bl. bissettiae*), PV683519-PV683520 (*Bl. californiensis*), PV683521 (*Bl. carolinensis*), PV683522-PV683526 (*B. miyamotoi*). The intergenic spacer sequences, in turn, have been given the following accession numbers: PV683527-PV683536 (*Bl. afzelii*), PV683537-PV683539 (*Bl. garinii*), PV683540-PV683543 (*Bl. burgdorferi*), PV683544-PV683546 (*Bl. valaisiana*), PV683547-PV683550 (*Bl. spielmanii*), PV683551-PV683553 (*Bl. bissettiae*), PV683554-PV683555 (*Bl. californiensis*), PV683556 (*Bl. carolinensis*), PV683557-PV683561 (*B. miyamotoi*).

The obtained sequences were compared to the reference sequences for the individual species identified in the presented studies, i.e., *Bl. afzelii* PKo (CP002933), *Bl. garinii* 20047 (CP018744), *Bl. burgdoferi* PAli (CP019844), *Bl. valaisiana* VS116 (ABCY02000001), *Bl. spielmanii* Pmew (CP124042), *Bl. bissettiae* Pgeb (CP124109), *Bl. californiensis* CA443 (CP124076), *Bl. carolinensis* SCW-22 (CP132465), *B. miyamotoi* ZStruIII14-9 (CP114720). The aligned sequences representing the *flaB* gene fragments and *mag-trnI* intergenic region mentioned above were analyzed using the MEGA11 (Molecular Evolutionary Genetics Analysis, version 11) software [49]. Relationships between individual sequences within each marker analyzed (*flaB* or *mag-trnI*) were determined using the genetic distances between sequences as a measure of the number of nucleotide substitutions for the loci analyzed. They represented the ratio of the number of distinct nucleotides to the total number of nucleotides in the sequence analyzed. Genetic distance values were measured as the average distance within each group of sequences representing a separate species (as a measure of intraspecies variation) and as the average interspecies distance (as a measure of variation between each pair of species being compared). Genetic distances were calculated according to Tamura’s 3-parametric model using the maximum likelihood (ML) method with 1000 bootstrap replicates [49].

**Statistical analysis.** The chi-squared test with the Yates correction was used to perform statistical analyses. The following data were analyzed: the proportion of individual tick stages attacking humans in individual years and in individual provinces; the proportions of ticks with varying degrees of engorgement (engorged, partially engorged, unfed) within individual stages and between tick stages; the level of tick infection depending on the stage, year, province and degree of engorgement of the tick; and the spread of *Borreliaceae* species depending on the stage of the tick, year of detection, province and degree of engorgement of the tick. The analyses mentioned above involved multiple testing; therefore, the Benjamini–Hochberg correction was applied to adjust the false discovery rate, and *p* values < 0.05 were considered statistically significant. All calculations were performed using the Statistica 13.0 software (StatSoft Inc., Tulsa, OK, USA).

## 3. Results

### 3.1. Identification of Tick Species Attacking Humans

Of the 1232 ticks collected from humans during the study period from 2018 to 2022, the majority, namely 1194 (96.9%), were *I. ricinus*, while the remaining 38 (3.1%) belonged to *D. reticulatus* (Table 2).

Of the 1194 individuals of *I. ricinus* attacking humans, 87 (7.3%) were larvae, 903 (75.6%) belonged to nymphs, 197 (16.5%) were classified as females and seven (0.6%) were males (Table 2). The highest number of ticks, 395, was collected in 2019; lower abundances were recorded in 2020 and 2021 (247 and 224 individuals, respectively) and the lowest in 2018 and 2022 (161 and 167 individuals, respectively; Table 2). In subsequent years, the proportion of individual stages ranged from 3.6% to 12.1% for larvae, from 70.2% to 79.2% for nymphs and from 12.9% to 22.7% for adults (Table 2), with statistically significant differences occurring only in the proportion of larvae when comparing 2020 and 2021 (*p* = 0.02709).

*Ixodes ricinus* ticks obtained for testing came from all sixteen provinces of Poland (Figure S1). The largest number, 344 ticks, was obtained from Zachodniopomorskie Province due to the ease of delivering samples for testing, and the smallest number of samples for testing, 39, came from Kujawsko-Pomorskie Province (Figure S1, Table 3). A comparison of the proportions of individual stages in all provinces did not reveal any statistically significant differences (*p* > 0.06705).

**Figure 1:**
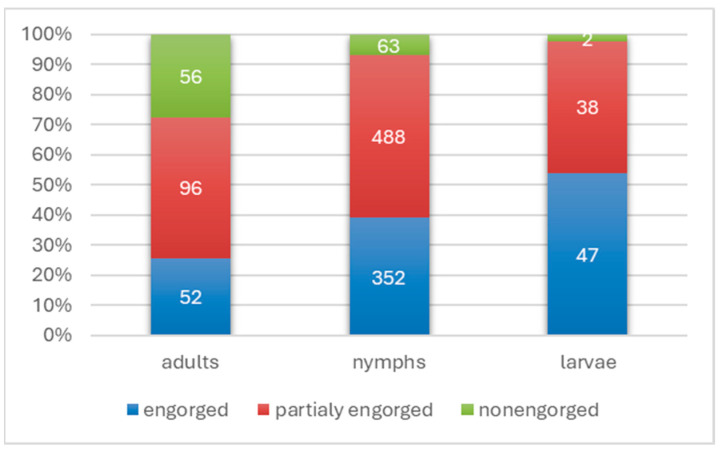
The rates of engorgement of *I. ricinus* ticks obtained from humans.

Of all *I. ricinus* specimens, as many as 1073 (90%) were ticks in different stages of feeding, of which 622 (52.1% of all specimens) were partially engorged ticks. Among larvae, fully engorged individuals dominated (54%), with 39% among nymphs and 25.5% among adults (Figure 1), and the differences were statistically significant between all stages (*p* < 0.0064). On the other hand, the proportion of partially engorged specimens was 43.7% among larvae, while it was dominant among nymphs (54%) and adults (47.1%, Figure 1), but differences between stages were not statistically significant (*p* > 0.05195). The lowest percentage was recorded among non-engorged individuals and was 2.3% among larvae, 7% among nymphs and 27.4% among adults (Figure 1), while statistically significant differences did not occur between the values recorded among larvae and nymphs (*p* = 0.09248) but were observed when both stages were compared to adults (*p* < 0.00001).

**Figure 2:**
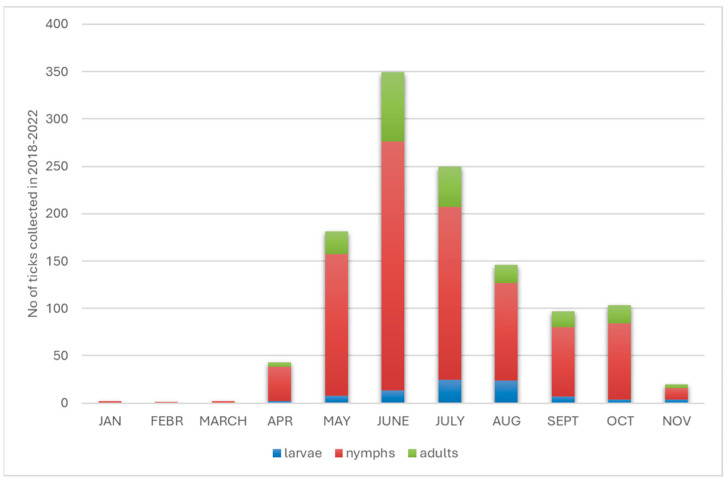
Seasonal activity of the different stages of *I. ricinus* ticks.

In individual years, ticks were active from January to November, but only single individuals were collected from January to March, while two peaks, characteristic of Central Europe, were recorded in tick activity, namely in June and October, but the latter peak was more than three times smaller (Figure 2). A comparison of the proportions of each stage of *I. ricinus* in individual months showed statistically significant differences only in the case of larvae when comparing May to August, June to July, June to August and August to October (*p* < 0.02768, Figure 2). For larvae, the median value was eight, with the lowest abundance of two larvae recorded in April and the highest (n = 25) in June. Moreover, the highest abundance of nymphs (n = 263) and imago (n = 73) was recorded in June, while the median by month for nymphs was 73 and that for adults was 19.

**Figure 3:**
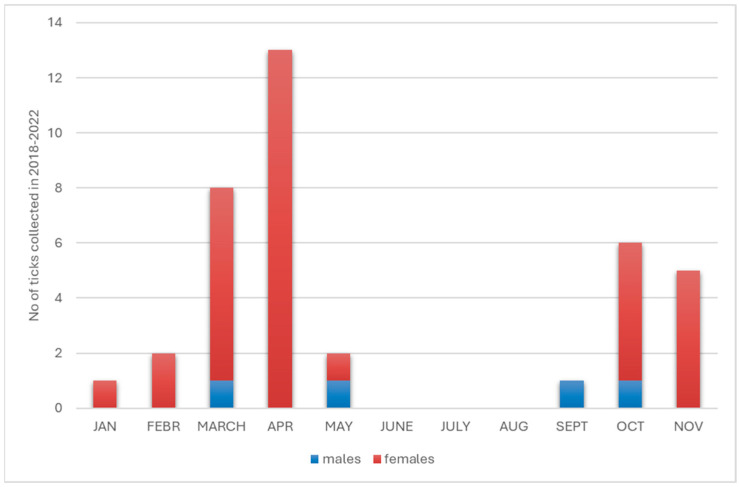
Seasonal activity of *D. reticulatus* ticks.

Within the species *D. reticulatus* attacking humans between 2018 and 2022, 38 individuals were found, of which 34 (89.5%) were females and four (10.5%) were males. The highest number of individuals was collected in 2021 (12; 31.6%), as well as in 2020 (10; 26.3%) and 2019 (9; 23.7%). Throughout the study period, the median number of ticks of this species collected per month was four; however, the highest activity was observed in March and April (8 and 13; 55.3% of all *D. reticulatus* individuals, respectively), while no individuals of this species were collected during the months of June to August (Figure 3). Attacks by *D. reticulatus* were recorded only in the Zachodniopomorskie and Lubuskie provinces (34 and 4 individuals, respectively; Table 3).

### 3.2. Prevalence of Borreliaceae Spirochetes in I. ricinus and D. reticulatus Ticks

Overall, the infection rate of *I. ricinus* ticks collected from humans with *Borreliaceae* spirochetes determined on the basis of nested PCR reactions was 18.3% (219/1194; Table 4).

The rates of tick infection by year ranged from 5.4% in 2022 to 27.5% in 2020, and the differences were statistically significant (*p* < 0.0397), except for years 2018/2021, 2018/2022 and 2019/2020 (*p* > 0.1305; Table 4). Statistically significant differences were not observed for adults (*p* > 0.1314) and larvae (*p* > 0.8269) but were recorded for nymphs in most of the years compared (*p* < 0.0189), with the exception of 2018/2021, 2018/2022, 2019/2020 and 2021/2022 (*p* > 0.086, Table 4). In contrast, statistically significant differences did not occur between the different stages in each year (*p* > 0.1896, Table 4).

*D. reticulatus* ticks removed from humans between 2018 and 2022 showed an overall infection rate of 34.2% (13/38; Table 4).

In individual provinces, the level of infection of *I. ricinus* ticks ranged from 10% (Opolskie) to 24.2% (Pomorskie), and the differences were not statistically significant for any province (*p* > 0.571, Table 5).

No statistically significant differences were also observed when comparing the infection rate in individual stages from different provinces (*p* > 0.1509), although they ranged from 0% to 33.3% for adults, from 10% to 27% for nymphs and from 0% to 50% for larvae (Table 5). Differences were also not observed when comparing the infection rate at different stages in individual provinces (*p* > 0.2538).

**Figure 4:**
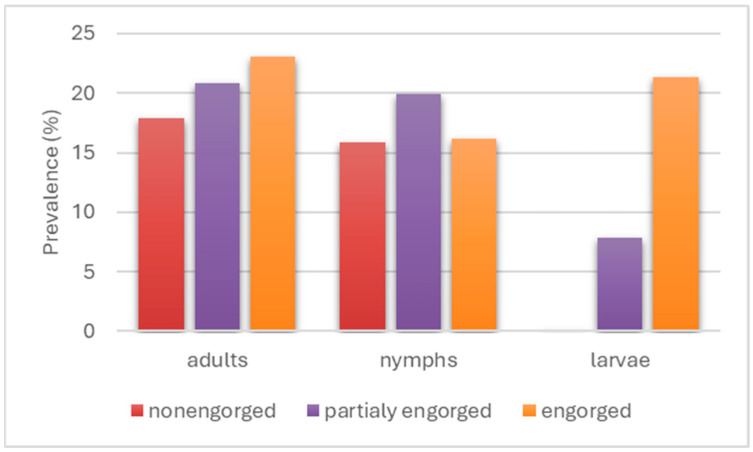
Infection of *I. ricinus* ticks with spirochetes of the *Borreliaceae* family in relation to the engorgement level.

An analysis of *I. ricinus* tick infection as a proportion of the degree of engorgement within each stage showed an increase in the infection rate with increasing degrees of engorgement in larvae, from 0% in non-engorged specimens through 7.9% in partially engorged specimens to 21.3% in fully engorged specimens and from 17.9% in non-engorged adults through 20.8% in partially engorged specimens to 23.1% in fully engorged specimens. In contrast, for nymphs, non-engorged individuals showed an infection rate of 15.9%, partially engorged individuals showed an infection rate of 21.9%, and fully engorged nymphs showed a decrease in the infection rate to 16.2% (Figure 4). A comparison of these results across developmental stages showed no statistically significant differences (*p* > 0.3489).

### 3.3. Identification of Borreliella and Borrelia Species in I. ricinus and D. reticulatus Ticks

The identification of spirochete species was based on the sequence length polymorphism present between the *mag* and *trnI* genes and on the restriction fragment length polymorphism present in the *flaB* gene sequence. The use of these two markers allowed the identification of spirochete species from the *Borreliaceae* family in all 232 positive samples, in which a total of nine spirochete species were detected. Among the positive samples, 104 (44.8%) were represented by *Bl. afzelii*; the next most detected species was *B. miyamotoi* (15.1%), followed by *Bl. burgdorferi* (14.2%), *Bl. valaisiana* and *Bl. spielmanii* (7.3% each), *Bl. garinii* (5.6%), *Bl. bissettiae* (4.3%), *Bl. californiensis* (0.9%) and *Bl. carolinensis* (0.4%, Table 6).

The distribution of *Borreliaceae* species within the different developmental stages of *I. ricinus* ticks and in *D. reticulatus* females showed no statistically significant differences (*p* > 0.5462). In the case of *I. ricinus*, the adults were more frequently infected with *Bl. afzelii* (35.7%), *Bl. burgdorferi* (23.8%) and *B. miyamotoi* (19%) than by other species (Table 6). Similarly, *Bl. afzelii* (47.6%), *Bl. burgdorferi* (13.4%) and *B. miyamotoi* (12.8%) were more frequently detected in nymphs, while *Bl. afzelii* (46.2%) and *Bl. spielmanii* (23.1%, Table 6) predominated in larvae. Females of *D. reticulatus*, on the other hand, were predominantly infected with *Bl. afzelii* and *B. miyamotoi* (38.5% each).

**Figure 5:**
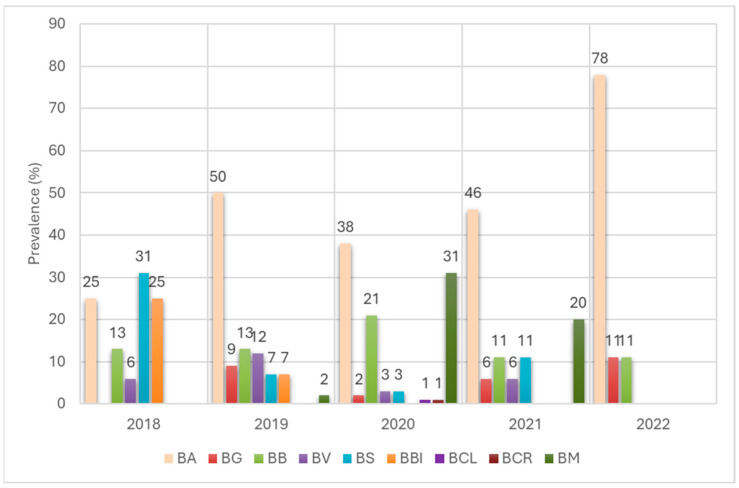
Occurrence of *Borreliaceae* species in *I. ricinus* ticks removed from humans between 2018 and 2022. BA—*Bl. afzelii*, BG—*Bl. garinii*, BB—*Bl. burgdorferi*, BV—*Bl. valaisiana*, BS—*Bl. spielmanii*, BBI—*Bl. bissettiae*, BCL—*Bl. californiensis*, BCR*—Bl. carolinensis*, BM—*B. miyamotoi*.

The distribution of individual spirochete species in *I. ricinus* ticks varied from year to year (Figure 5). In all years of the study, only two species were detected, namely *Bl. afzelii* and *Bl. burgdorferi*. The species *Bl. afzelii* dominated in all years from 2019 onwards (infection ranging from 38% of infected ticks in 2020 to 78% in 2022), except for 2018 (25%), when *Bl. spielmanii* was the dominant species (31%). Infection with the species *Bl. burgdorferi* ranged from 11% of infected ticks in 2021 and 2022 to 21% in 2020.

The species *Bl. spielmanii* was detected in four consecutive years from 2018 to 2021 (infection from 3% of infected ticks in 2020 to 31% in 2018), as was *Bl. valaisiana* (from 3% in 2020 to 12% in 2019), while *Bl. garinii* was also detected in four consecutive years but from 2019 to 2022 (from 2% in 2020 to 11% in 2022). Infection with *B. miyamotoi* species was detected in three consecutive years from 2019 to 2021 and ranged from 2% in 2019 to 31% in 2020. *Bl. bissettiae* was detected only in 2018 (25%) and 2019 (7%), while the *Bl. californiensis* and *Bl. carolinensis* species were detected only in 2020 and both accounted for 1% of infected ticks each (Figure 5).

The distribution of individual species of *Borreliaceae* spirochetes in ticks attacking humans in individual provinces was also compared. In *I. ricinus* ticks, the only species present in all provinces was *Bl. afzelii*. *B. miyamotoi* was detected in 13 provinces; *Bl. burgdorferi* in 11; *Bl. garinii*, *Bl. valaisiana* and *Bl. spielmanii* in seven; *Bl. bissettiae* in three; and *Bl. californiensis* and *Bl. carolinensis* in individual provinces (Table S1). The province with the highest number of detected species was Zachodniopomorskie (eight species, except *Bl. californiensis*), seven species were detected in Śląskie Province (except *Bl. bissettiae* and *Bl. carolinensis*), six in Podkarpackie Province, and five in Dolnośląskie Province. The lowest numbers of species were detected in Lubuskie and Mazowieckie (three each), Opolskie (two) and Kujawsko-Pomorskie (one), and, in the remaining seven provinces, four species of *Borreliaceae* spirochetes were found (Table S1). The species *Bl. afzelii* dominated in 14 provinces, where it accounted for 30% to 100% of infected ticks, with the exception of Małopolskie Province, where *B. miyamotoi* dominated (45.5% of infected ticks), and Podlaskie Province, where *Bl. burgdorferi* was the dominant species (42.9% of infected ticks, Table S1). The differences in the infection rates of ticks with individual species of *Borreliaceae* spirochetes were not statistically significant in any of the provinces studied (*p* > 0.1585).

**Figure 6:**
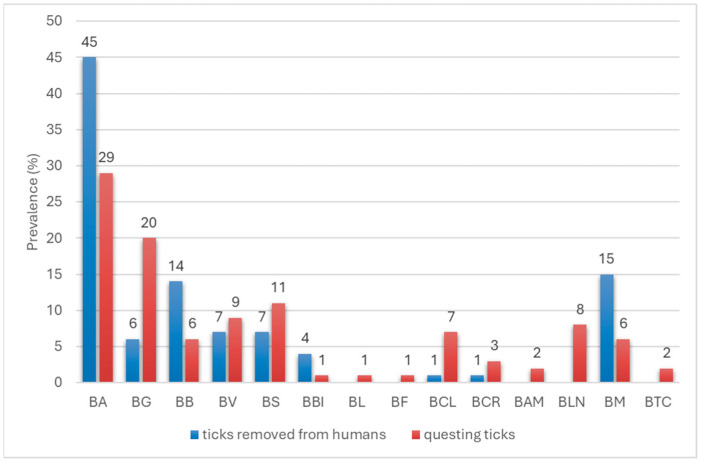
Comparison of the prevalence of spirochete species of the family *Borreliaceae* in *I. ricinus* ticks removed from humans in 2018–2022 and those collected from vegetation in our previous studies [9]. BA—*Bl. afzelii*, BG—*Bl. garinii*, BB—*Bl. burgdorferi*, BV—*Bl. valaisiana*, BS—*Bl. spielmanii*, BBI—*Bl. bissettiae*, BL—*Bl. lusitaniae*, BF—*Bl. finlandensis*, BCL—*Bl. californiensis*, BCR—*Bl. carolinensis*, BAM—*Bl. americana*, BLN—*Bl. lanei*, BM—*B. miyamotoi*, BTC—*B. turcica*.

A comparison of the species diversity of *Borreliaceae* spirochetes in ticks removed from humans and those collected from vegetation in our previous study [9] showed that ticks removed from humans were more frequently infected by the species *Bl. afzelii*, *Bl. burgdorferi*, *Bl. bissettiae* and *B. miyamotoi* than ticks collected from vegetation (*p* < 0.01606), while *Bl. garinii* and *Bl. californiensis* were significantly more common in host-seeking ticks (*p* < 0.00044, Figure 6). Of the spirochete species detected only in host-seeking ticks, only *Bl. lanei* showed a statistically significant difference in the infection rate (*p* = 0.000145, Figure 6).

**Figure 7:**
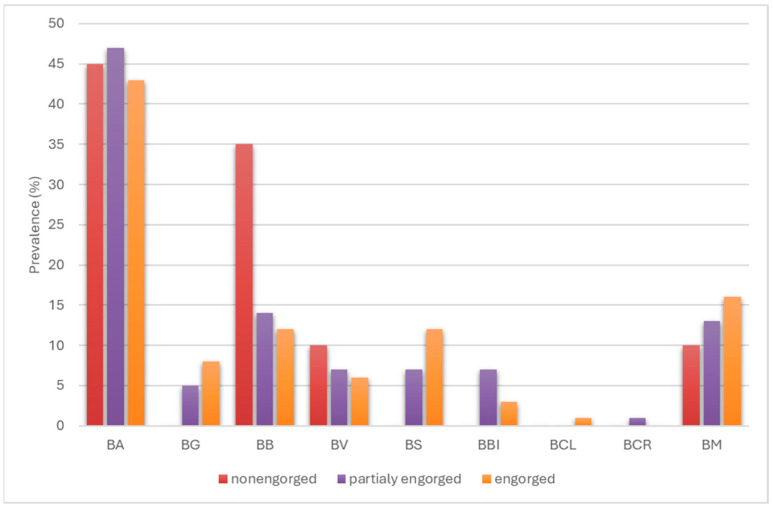
Comparison of infection rates of *I. ricinus* ticks with species of the *Borreliaceae* family according to their engorgement status. BA—*Bl. afzelii*, BG—*Bl. garinii*, BB—*Bl. burgdorferi*, BV—*Bl. valaisiana*, BS—*Bl. spielmanii*, BBI—*Bl. bissettiae*, BCL—*Bl. californiensis*, BCR—*Bl. carolinensis*, BM—*B. miyamotoi*.

The tick infection rates of individual species of spirochetes of the *Borreliaceae* family were also compared in relation to the degree of tick engorgement (Figure 7).

For three species, there was an increase in the percentage of tick infection with an increase in the degree of tick engorgement, namely *Bl. garinii*, *Bl. spielmanii* and *B. miyamotoi*, but the differences were not statistically significant (*p* > 0.5098). Two species, *Bl. burgdorferi* and *Bl. valaisiana*, showed a decrease in infection rate with an increase in the degree of tick engorgement, and statistically significant differences were recorded for *Bl. burgdorferi* when comparing the infection rate of non-engorged ticks to that of fully engorged ticks (*p* = 0.03936). In contrast, for *Bl. afzelii* and *Bl. bissettiae*, there was an increase in the infection rate when ticks were partially engorged and a decrease when ticks were fully engorged, while differences in infection rates were not statistically significant (*p* > 0.8862).

**Figure 8:**
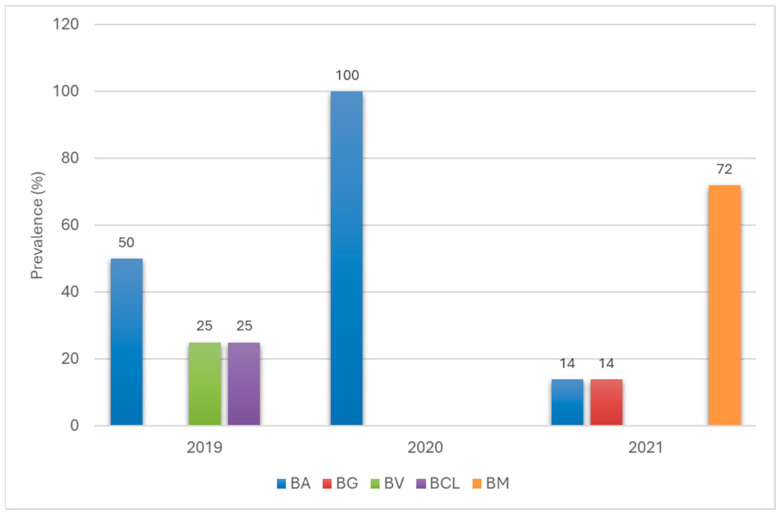
Occurrence of *Borreliaceae* spirochete species in *D. reticulatus* ticks removed from humans in 2019–2021. BA—*Bl. afzelii*, BG—*Bl. garinii*, BB—*Bl. burgdorferi*, BV—*Bl. valaisiana*, BCL—*Bl. californiensis*, BM—*B. miyamotoi*.

In the case of *D. reticulatus* ticks, infection with spirochetes of the *Borreliaceae* family was detected only in the years 2019 to 2021 (Figure 8). Of the five *Borreliaceae* species detected in *D. reticulatus*, the only species detected in all these years was *Bl. afzelii*, which dominated in 2019 (50%) and was the only one detected in 2020, while, in 2021, *B. miyamotoi* dominated (72%), and *Bl. afzelii* and *Bl. garinii* were present in 14% of *D. reticulatus* each. The species *B. miyamotoi* and *Bl. garinii* were detected only in 2021, while *Bl. valaisiana* and *Bl. californiensis* were detected only in 2019 (both accounting for 25% of infected ticks each; Figure 8).

The distribution of *Borreliaceae* species in *D. reticulatus* ticks was limited to only two provinces from which this tick species was collected. All five species of *Borreliaceae* detected in *D. reticulatus* were found in individuals from Zachodniopomorskie Province, i.e., *Bl. afzelii*, *B. miyamotoi*, *Bl. garinii*, *Bl. valaisiana* and *Bl. californiensis*, although the latter was not detected in *I. ricinus* ticks from this province (Table S1). The species identified in the only infected individual of *D. reticulatus* from Lubuskie Province was *B. miyamotoi* (Table S1).

### 3.4. Genetic Variability of Borreliaceae Detected in Ticks Removed from Humans

An analysis of the DNA sequences of the *mag*-*trnI* and *flaB* markers obtained for samples representing each identified species confirmed the validity of the identification as determined by the sequence length polymorphism occurring between the *mag* and *trnI* genes and the restriction fragment length polymorphism within the *flaB* gene sequence. For the *mag-trnI* intergenic region, the mean genetic distance within species of the genus *Borreliella* ranged from 0.0017 for *Bl. californiensis* to 0.0093 for *Bl. burgdorferi*, and it was 0.0021 for *B. miyamotoi* (Table S2). In contrast, the genetic distance between species of the genus *Borreliella* ranged from 0.0868 for *Bl. bissettiae* and *Bl. carolinensis* to 0.2818 for *Bl. bissettiae* and *Bl. spielmanii* (Table S3). The genetic distance between species belonging to the genera *Borreliella* and *Borrelia* ranged from 0.5966 for *Bl. californiensis* and *B. miyamotoi* to 0.712 for *Bl. spielmanii* and *B. miyamotoi* (Table S3).

A comparison of the *flaB* gene sequences showed genetic distance values within *Borreliella* species ranging from 0.009 for *Bl. afzelii* to 0.081 for *Bl. garinii*, and it was 0 for *B. miyamotoi* (Table S4). The genetic distance values between *Borreliella* species ranged from 0.0067 for *Bl. bissettiae* and *Bl. carolinensis* to 0.0668 for *Bl. garinii* and *Bl. burgdorferi* (Table S5). The genetic distance values between species belonging to the genera *Borreliella* and *Borrelia* ranged from 0.1693 for *Bl. valaisiana* and *B. miyamotoi* to 0.1863 for *Bl. bissettiae* and *B. miyamotoi* (Table S5).

## 4. Discussion

Lyme borreliosis (LB) is one of the most problematic tick-borne diseases in the northern hemisphere. Assessing the spirochete prevalence is essential in evaluating the LB risk [50]. Our study of the occurrence of *Borreliaceae* spirochetes in two species of ticks attacking humans in Poland over five consecutive years, 2018–2022, provides a picture of the risk of acquiring LB but also Borrelia miyamotoi disease (BMD), which is also diagnosed in humans after tick contact [2]. Testing of ticks removed from humans after tick bites for the risk of acquiring the aforementioned spirochetes is not performed very often in Europe [26,27,28,29,30,31,32,33,34,35,51], including Poland [36,52].

The tick species collected in our study were *I. ricinus*, mainly associated with deciduous and mixed forest areas, and *D. reticulatus* preferring more exposed meadow areas and forest edges [53]. The latter species was represented in our study by the adult stage, mainly females, and showed two peaks in activity typical for this species [54]: winter–spring from January to May and autumn from September to November. *Dermacentor reticulatus* is the second most common tick species in Europe after *I. ricinus* and can also sporadically attack humans [30,33,34,35]. Females of *D. reticulatus* were infected by four species of the genus *Borreliella* (mainly *Bl. afzelii*, but also *Bl. garinii*, *Bl. valaisiana* and *Bl. californiensis*) and by *B. miyamotoi*. This might suggest a potential role for *D. reticulatus* in the spread of these species, as different species of the genus *Borreliella* and *B. miyamotoi* are also detected in host-seeking *D. reticulatus* [37,39], as well as in ticks feeding on deer and dogs [41,42]. However, the relatively small number of ticks of this species examined in this study means that the results should be treated with caution and not considered definitive. In contrast to *D. reticulatus*, the activity of *I. ricinus* peaked in June, with nymphs predominating—consistent with prior studies [33,34,35,36].

In the last decade, across Europe, including Poland, the level of *Borreliaceae* spirochete infection in *I. ricinus* ticks attacking humans has ranged from 8.7% to 29% [30,31,32,33,34,35,36,51,52,55]. In our study, the average overall infection rate of this tick species with *Borreliaceae* spirochete species was 18.3%, but the level of infection fluctuated in different years, increasing from 9.9% in 2018 to 27.5% in 2020 and then decreasing to 5.4% in 2022. In contrast to this phenomenon, the number of recorded cases of LB in Poland increased in the years 2018 and 2019 and then decreased during the first two years of the COVID-19 pandemic (2020–2021) to rise again in 2022. The two phenomena therefore do not appear to be directly related, and the reported decline in infections among ticks affecting humans may be due to the influence of different but related environmental factors, especially climatic factors such as temperature and humidity, which shape the tick population size and density and also affect the survival, abundance and availability of tick hosts, which are also the reservoirs of spirochetes of the *Borreliaceae* family [25]. This thesis is supported by regional differences in the level of *I. ricinus* infection, which ranged from 10% to 24.2% depending on the province. The level of infection of ticks removed from humans in our study was similar to that found in earlier studies of ticks collected from vegetation, taking into account their local diversity [9].

The level of *Borreliaceae* spirochetes infection of the different stages of ticks removed from humans in our study was the lowest in larvae and highest in the mature stage, which was consistent with previous data on the infection of ticks collected from vegetation [9], as well as with data from other studies of ticks collected from humans [26,27,28,30,36,52,55]. This is also consistent with the life cycle of the *I. ricinus* tick, which feeds only once per developmental stage, and the limited transovarian transmission capacity of *Borreliella* spirochetes, resulting in the lowest infection rate among larvae [8]. Unlike species of the genus *Borreliella*, *Borrelia* spirochete species show the capacity for transovarial transfer, including species carried by ticks of the family Ixodidae, such as *B. miyamotoi* spread in Europe by *I. ricinus* [18,56]. The detection of *B. miyamotoi* spirochetes in *I. ricinus* larvae in our study confirms the possibility of transovarial transfer, as do other studies, whether in ticks acquired from humans [32,36,55] or collected from hosts [44] or from vegetation [9]. However, the possibility of mammalian infection by transovarial infected larvae has only been observed in *I. scapularis* occurring in North America [57].

Of the 14 *Borreliella* species spread in Europe by *Ixodes* ticks, some—in particular, *Bl. afzelii*, *Bl. garinii* and *Bl. burgdorferi*—can cause various symptoms associated with LB; in addition, this tick also transmits *B. miyamotoi*, causing BMD. Due to the multitude of spirochete species carried by *I. ricinus*, it is important to precisely identify the spirochete species involved in the infection. This accuracy is ensured by the dual-marker approach used in our research. In contrast, other analyses of spirochete species detected in ticks removed from humans showed lower species identification efficiencies for the genus *Borreliella* and the need to use separate primers for *Borrelia* DNA detection, particularly *B. miyamotoi* [26,28,29,30,33,34,35,36]. Furthermore, certain species, such as *Bl. bavariensis*, *Bl. spielmanii*, *Bl. valaisiana*, *Bl. lusitaniae* or *Bl. bissettiae*, cause less specific symptoms, often of a mixed nature, compared to *Bl. afzelii*, *Bl. garinii* and *Bl. burgdorferi*, and species hitherto considered non-pathogenic to humans, such as *Bl. californiensis* and *B. turcica*, are also detected in symptomatic individuals with LB [11]. This indicates the need for diagnostic tests not limited to *Bl. afzelii*, *Bl. garinii* and *Bl. burgdorferi*, as, in many cases with LB symptoms, this may lead to obtaining false negative results and consequently a lack of treatment.

Among the nine species of the family *Borreliaceae* detected in our study, *Bl. afzelii* was consistently the most prevalent species, in line with prior European studies concerning ticks collected from humans [26,27,28,29,30,31,32,33,34,35,36,51,52,55,58] and those collected from vegetation [9,14,16,59]. The second most common species was *B. miyamotoi*, which was slightly behind *Bl. burgdorferi*. Both species were significantly more frequent in ticks collected from humans than in natural tick populations studied previously [9], in which *Bl. garinii* and *Bl. spielmanii* predominated, in addition to *Bl. afzelii*. The latter species was much less frequent in ticks collected from humans than from vegetation, as were *Bl. garinii* and *Bl. valaisiana*. The least frequent species detected in ticks collected from humans were *Bl. bissettiae*, *Bl. californiensis* and *Bl. carolinensis*, but *Bl. bissettiae* occurred more frequently than in ticks from vegetation, and *Bl. californiensis* and *Bl. carolinensis* occurred less frequently. Differences in the prevalence of particular *Borreliaceae* species in human-feeding and host-seeking ticks may be due to the variations in the environments within which ticks attack humans, namely the availability of tick hosts, which constitute a diverse reservoir for individual spirochete species, but also the relative density of the tick population [60]. Of the spirochete species more frequently detected in ticks feeding on humans, *Bl. afzelii* is particularly associated with mammals, especially small and medium-sized mammals, as is *Bl. spielmanii*, but also *Bl. burgdorferi*, whose reservoir consists of both mammals and birds [13]. In contrast, species preferring only an avian reservoir, such as *Bl. garinii* and *Bl. valaisiana*, may, as in our study, occur much less frequently in ticks attacking humans than in natural populations collected from vegetation [28,30,36]. When comparing the level of infection of *I. ricinus* ticks with *Borreliella* spirochaetes depending on the degree of engorgement, a proportional increase in its rate was found only for *Bl. garinii* and *Bl. spielmanii*, which, in the case of the latter, would confirm the association of the species with a mammalian reservoir, with their more frequent detection in ticks attacking humans. In turn, this thesis is confirmed for ticks infected with *Bl. garinii* among specimens attacking humans by the generally low percentage of their infection. Confirmation of this thesis is supported by the fact that the percentage of infections with *Bl. burgdorferi* and *Bl. valaisiana*—species associated partly or exclusively with birds—was inversely proportional to the degree to which ticks were engorged. Although *Bl. burgdorferi* was detected more frequently in ticks obtained from humans than from vegetation, the highest percentage was recorded in non-engorged ticks. This phenomenon is also confirmed by the percentage of infections with *Bl. afzelii* species, varying only slightly—independently of the degree of tick engorgement—found in our study.

Among the spirochete species spread by *I. ricinus*, the only well-documented representative of the genus *Borrelia* is *B. miyamotoi*, causing BMD. It is a species that is regularly recorded in Europe, including Poland, but with a relatively low frequency in natural tick populations [9,18,61,62]. This species is also detected in ticks attacking humans [26,29,30,31,32,33,35,36,52,55,58]. However, in our study of ticks feeding on humans, it was much more frequently detected than in ticks collected from vegetation [9], similarly to other studies conducted in Poland [32] but different from studies in neighboring Germany [30]. The species showed significant variation in the level of occurrence in infected ticks between years and was only detected in 2019–2021, when the highest tick infection rates were recorded. In European studies, the species was detected in ticks collected from birds and mammals and in birds and mammals themselves, indicating a potentially wide reservoir range [44,63,64,65]. To date, xenodiagnostic studies have confirmed the potential for *B. miyamotoi* to act as a reservoir for *Apodemus flavicollis* and *Myodes glareolus* only [66]. Studies of tick populations from different areas of Europe testify to a local (‘insular’) distribution within individual European countries, which would confirm the involvement of small mammals as a reservoir for this spirochete species [9,62,63]. Our results showing a proportional increase in the infection rates of *B. miyamotoi* with increasing tick engorgement support these findings.

The multitude of *Borreliaceae* species spread by *I. ricinus* is associated with the possibility of co-infection with several species simultaneously in ticks, and this implies a risk of human exposure to multiple infections. Tick co-infection occurs primarily through the ability of *I. ricinus* to feed on multiple vertebrate species that are reservoirs of various *Borreliella* species and *B. miyamotoi*. The mechanism for acquiring several species of bacteria results from a combination of vertical (transovarial) and horizontal (blood-taken) transfer, or simultaneous systemic infection (from the host) and co-infection with another (infected) tick at the same time, as well as the intermittent feeding of larvae on different infected hosts [56,67]. Because co-infection requires confluence among several circumstances, its reported incidence varies from 0.9% to 31.5% in host-seeking ticks [9,38,59,68,69,70] and from 4.2% to 33% in host-harvested ticks [12,41,42,43,71,72]. In contrast, this phenomenon was not found in our study, as in many other studies [26,27,29,33,34,35,51,55]. However, this phenomenon also occurs in studies of ticks obtained from humans and in a highly variable range, from 0.8% to 25%, with the rates in studies from Poland not exceeding 2.7% [28,30,31,32,36,52,58]. Our study therefore confirms the low risk of multiple infections in humans in Poland.

## 5. Conclusions

Our study confirms the high risk of *Borreliaceae* infection for humans in Poland after a tick bite with *I. ricinus*, which is the typical vector of these bacteria, but not necessarily for *D. reticulatus*, the second most common tick species in Poland, due to the small number of individuals examined. Despite annual fluctuations, the main concern is *Bl. afzelii*, the most common species in Europe, including Poland, while two others, *Bl. burgdorferi* and *B. miyamotoi*, may also pose a real risk and should not be underestimated. Studies have shown that the overall risk of infection increases with the degree of tick engorgement, but, in general, this correlation applies only to certain species of spirochetes associated with mammals as reservoirs. Due to the overwhelming proportion of engorged ticks collected from humans, it is essential to make people aware of the necessary prophylaxis against tick attacks. Accurate knowledge of the prevalence and distribution of spirochete species of the *Borreliaceae* family is an important tool in assessing the risk of acquiring diseases caused by these bacteria.

## Figures and Tables

**Table 1:** Primers used for DNA amplification of *Borreliaceae* spirochetes.

Genetic Marker	Sequence of Primers (5′->3′)	Annealing Temp. (°C)	Length of Amplicons (bp)	Usage	Reference
*flaB*	FL84F: AGAAGCTTTCTAGTGGGTACAGA FL976R: GATTGGCCTGTGCAATCAT	57	893	PCR-RFLP, sequencing	[7]
	Nested PCR				
	220f: CAGACAACAGAGGGAAAT				
	823r: TCAAGTCTATTTTGGAAAGCACC	54	604	PCR-RFLP	[43]
	FL120F: TGATGATGCTGCTGGGATGG				
	FL908R: TCATCTGTCATTGTAGCATCTT	56	789	Sequencing	[7]
*mag-trnI*	mag-268F: TCTAATTAAAACAGCHTGDGGAYT				
	trnI-20R: TGAACATCCGACCTCAGG	51	521–1395		[41]
	Nested PCR			PCR-SLP, sequencing	
	mag-435F: CCATATAAGCTTCCGTTTCAAC				
	trnI-65R: CTAACCACCTGAGCTATGATCC	51	309–1183	PCR-SLP, sequencing	[41]

**Table 2:** Activity of *Ixodes ricinus* and *Dermacentor reticulatus* ticks attacking humans between 2018 and 2022.

Year of Study	Stage/Sex (%)				Total
	Females	Males	Nymphs	Larvae	
*Ixodes ricinus*					
2018	29 (18)	2 (1.2)	113 (70.2)	17 (10.6)	161
2019	57 (14.5)	2 (0.5)	313 (79.2)	23 (5.8)	395
2020	55 (22.3)	1 (0.4)	182 (73.7)	9 (3.6)	247
2021	27 (12.1)	2 (0.8)	168 (75)	27 (12.1)	224
2022	29 (17.4)		127 (76)	11 (6.6)	167
Subtotal	197 (16.5)	7 (0.6)	903 (75.6)	87 (7.3)	1194
*Dermacentor reticulatus*					
2018	1 (100)				1
2019	8 (88.9)	1 (11.1)			9
2020	8 (80)	2 (20)			10
2021	12 (100)				12
2022	5 (83.3)	1 (16.7)			6
Subtotal	34 (89.5)	4 (10.5)			38
Total	231 (18.7)	11 (0.9)	903 (73.3)	87 (7.1)	1232

**Table 3:** Activity of *Ixodes ricinus* and *Dermacentor reticulatus* ticks attacking humans in different provinces of Poland.

Province	Stage/Sex (%)				Total
	Females	Males	Nymphs	Larvae	
*Ixodes ricinus*					
Zachodniopomorskie	54 (15.7)	6 (1.7)	238 (69.2)	46 (13.4)	344
Lubuskie	13 (19.2)	0 (0)	53 (77.9)	2 (2.9)	68
Wielkopolskie	11 (16.2)	0 (0)	52 (76.5)	5 (7.3)	68
Dolnośląskie	16 (16)	0 (0)	75 (75)	9 (9)	100
Opolskie	6 (15)	0 (0)	33 (82.5)	1 (2.5)	40
Pomorskie	12 (18.2)	0 (0)	52 (78.8)	2 (3)	66
Kujawsko-Pomorskie	9 (23.1)	0 (0)	30 (76.9)	0 (0)	39
Łódzkie	6 (14)	0 (0)	37 (86)	0 (0)	43
Śląskie	11 (17.4)	0 (0)	50 (79.4)	2 (3.2)	63
Warmińsko-Mazurskie	10 (20.8)	0 (0)	38 (79.2)	0 (0)	48
Mazowieckie	9 (18)	0 (0)	39 (78)	2 (4)	50
Świętokrzyskie	9 (16.1)	0 (0)	44 (78.6)	3 (5.3)	56
Małopolskie	12 (15.2)	0 (0)	52 (65.8)	15 (20)	79
Podlaskie	4 (10)	0 (0)	36 (90)	0 (0)	40
Lubelskie	8 (17.8)	0 (0)	37 (82.2)	0 (0)	45
Podkarpackie	7 (15.6)	1 (2.2)	37 (82.2)	0 (0)	45
Subtotal	197 (16.5)	7 (0.6)	903 (75.6)	87 (7.3)	1194
*Dermacentor reticulatus*					
Zachodniopomorskie	31 (91.2)	3 (8.8)	0 (0)	0 (0)	34
Lubuskie	3 (75)	1 (25)	0 (0)	0 (0)	4
Subtotal	34 (89.5)	4 (10.5)			38
Total	231 (18.7)	11 (0.9)	903 (73.3)	87 (7.1)	1232

**Table 4:** Infection of ticks with spirochetes of the *Borreliaceae* family in 2018–2022 by developmental stage and sex.

Year of Study	Stage/Sex [N/n (%)]				Total [N/n (%)]
	Females	Males	Nymphs	Larvae	
*Ixodes ricinus*					
2018	3/29 (10.3)	0/2 (0)	11/113 (9.7)	2/17 (11.8)	16/161 (9.9)
2019	14/57 (24.6)	0/2 (0)	71/313 (22.7)	6/23 (26.1)	91/395 (23)
2020	14/55 (25.5)	0/1 (0)	54/182 (29.7)	0/9 (0)	68/247 (27.5)
2021	8/27 (29.6)	1/2 (50)	21/168 (12.5)	5/27 (18.5)	35/224 (15.6)
2022	2/29 (6.9)		7/127 (5.5)	0/11 (0)	9/167 (5.4)
Subtotal	41/197 (20.8)	1/7 (14.3)	164/903 (18.2)	13/87 (14.9)	219/1194 (18.3)
*Dermacentor reticulatus*					
2018	0/1 (0)				0/1 (0)
2019	4/8 (50)	0/1 (0)			4/9 (44.4)
2020	2/8 (25)	0/2 (0)			2/10 (20)
2021	7/12 (58.3)				7/12 (58.3)
2022	0/5 (0)	0/1 (0)			0/6 (0)
Subtotal	13/34 (38.2)	0/4 (0)			13/38 (34.2)
Total	54/231 (23.4)	1/11 (9.1)	164/903 (18.2)	13/87 (14.9)	232/1232 (18.8)

N—number of infected; n—number of tested.

**Table 5:** Infection of ticks with spirochetes of the *Borreliaceae* family by developmental stage and sex in different provinces of Poland.

Province	Stage/Sex [N/n (%)]				Total
	Females	Males	Nymphs	Larvae	
*Ixodes ricinus*					
Zachodniopomorskie	14/54 (25.9)	1/6 (16.7)	49/238 (20.6)	9/46 (19.6)	73/344 (21.2)
Lubuskie	3/13 (23.1)	0 (0)	8/53 (15.1)	1/2 (50)	12/68 (17.6)
Wielkopolskie	2/11 (18.2)	0 (0)	8/52 (15.4)	0/5 (0)	10/68 (14.7)
Dolnośląskie	4/16 (25)	0 (0)	16/75 (21.3)	1/9 (11.1)	21/100 (21)
Opolskie	0/6 (0)	0 (0)	4/33 (12.1)	0/1 (0)	4/40 (10)
Pomorskie	4/12 (33.3)	0 (0)	11/52 (21.2)	1/2 (50)	16/66 (24.2)
Kujawsko-Pomorskie	1/9 (11.1)	0 (0)	3/30 (10)	0 (0)	4/39 (10.3)
Łódzkie	0/6 (0)	0 (0)	5/37 (13.5)	0 (0)	5/43 (11.6)
Śląskie	3/11 (27.3)	0 (0)	12/50 (24)	2 (3.2)	15/63 (23.8)
Warmińsko-Mazurskie	2/10 (20)	0 (0)	5/38 (13.2)	0 (0)	7/48 (14.6)
Mazowieckie	2/9 (22.2)	0 (0)	6/39 (15.4)	0/2 (0)	8/50 (16)
Świętokrzyskie	1/9 (11.1)	0 (0)	6/44 (13.6)	1/3 (33.3)	8/56 (14.3)
Małopolskie	4/12 (33.3)	0 (0)	7/52 (13.5)	0/15 (0)	11/79 (13.9)
Podlaskie	1/4 (25)	0 (0)	6/36 (16.7)	0 (0)	7/40 (17.5)
Lubelskie	0/8 (0)	0 (0)	8/37 (21.6)	0 (0)	8/45 (17.8)
Podkarpackie	0/7 (0)	0/1 (0)	10/37 (27)	0 (0)	10/45 (22.2)
Subtotal	41/197 (20.8)	1/7 (14.3)	164/903 (18.2)	13/87 (14.9)	219/1194 (18.3)
*Dermacentor reticulatus*					
Zachodniopomorskie	12/31 (38.7)	0/3 (0)	0 (0)	0 (0)	12/34 (35.3)
Lubuskie	1/3 (33.3)	0/1 (0)	0 (0)	0 (0)	1/4 (25)
Subtotal	13/34 (38.2)	0/4 (0)			13/38 (34.2)
Total	54/231 (23.4)	1/11 (9.1)	164/903 (18.2)	13/87 (14.9)	232/1232 (18.8)

N—number of infected; n—number of tested.

**Table 6:** Prevalence of *Borreliaceae* species in ticks removed from humans.

Spirochete Species	*Ixodes ricinus* (N/%)				*Dermacentor reticulatus* (Females Only; N/%)	Total (N/%)
	Stage			Subtotal		
	Adults	Nymphs	Larvae			
*Bl. afzelii*	15/35.7	78/47.6	6/46.2	99/45.2	5/38.5	104/44.8
*Bl. garinii*	2/4.8	9/5.5	1/7.7	12/5.5	1/7.7	13/5.6
*Bl. burgdorferi*	10/23.8	22/13.4	1/7.7	33/15.1		33/14.2
*Bl. valaisiana*	4/9.5	12/7.3		16/7.3	1/7.7	17/7.3
*Bl. spielmanii*	2/4.8	12/7.3	3/23.1	17/7.8		17/7.3
*Bl. bissettiae*	1/2.4	8/4.9	1/7.7	10/4.6		10/4.3
*Bl. californiensis*		1/0.6		1/0.5	1/7.7	2/0.9
*Bl. carolinensis*		1/0.6		1/0.5		1/0.4
*B. miyamotoi*	8/19	21/12.8	1/7.7	30/13.7	5/38.5	35/15.1
Total (N)	42	164	13	219	13	232

## Data Availability

All data generated or analyzed during this study are included in this published article and its Supplementary Materials. The accession numbers of DNA sequences obtained for bacteria are mentioned in Section 2 and are available in GenBank (https://www.ncbi.nlm.nih.gov/nuccore, accessed on 25 July 2025).

## Supplementary Materials

The following supporting information can be downloaded at https://www.mdpi.com/article/10.3390/pathogens14121234/s1. Figure S1. Number of ticks collected from humans in individual provinces in Poland in 2018–2022. The numbers in round brackets indicate the number of females/males/nymphs/larvae of Ixodes ricinus. The numbers in square brackets indicate the number of females/males of Dermacentor reticulatus. 1—Zachodniopomorskie Province, 2—Lubuskie Province, 3—Wielkopolskie Province, 4—Dolnośląskie Province, 5 Opolskie Province, 6—Pomorskie Province, 7—Kujawsko-pomorskie Province, 8—Łódzkie Province, 9—Śląskie Province, 10—Warmińsko-mazyrskie Province, 11—Mazowieckie Province, 12—Świętokrzyskie Province, 13—Małopolskie Province, 14—Podlaskie Province, 15—Lubelskie Province, 16—Podkarpackie Province. Table S1. Prevalence of Borreliaceae species in ticks removed from humans in different provinces of Poland. Table S2. *mag-trnI* intergenic spacer’s mean genetic distance within individual *Borreliaceae* species detected in host-seeking ticks from Northern Poland. Table S3. MEGA 11 results of mean distance between *Borreliaceae* species obtained on the basis of the intergenic spacer (IGS) of 3-methyladenine glycosylase (*mag*) and tRNA-Ile (*trnI*) gene sequence fragment comparison. Table S4. *flaB* gene mean genetic distance within individual *Borreliaceae* species detected in host-seeking ticks from Northern Poland. Table S5. MEGA 11 results of mean distance between *Borreliaceae* species obtained on the basis of *flaB* gene sequence fragment comparison.
