# Supplementary material for: Prevalence of Borreliaceae Spirochetes in Ticks Removed from Humans in Poland During 2018–2022
Source: Pathogens. 2025 Dec 3;14(12):1234. doi: 10.3390/pathogens14121234 (PMC12736064; doi:10.3390/pathogens14121234)
Supplement: Supplementary file 1 [file pathogens-14-01234-s001.zip › pathogens-3985952-supplementary.pdf]

Prevalence of *Borreliaceae* spirochetes in ticks removed from humans in Poland during 2018-2022

Beata Wodecka and Valentyna Kolomiiets

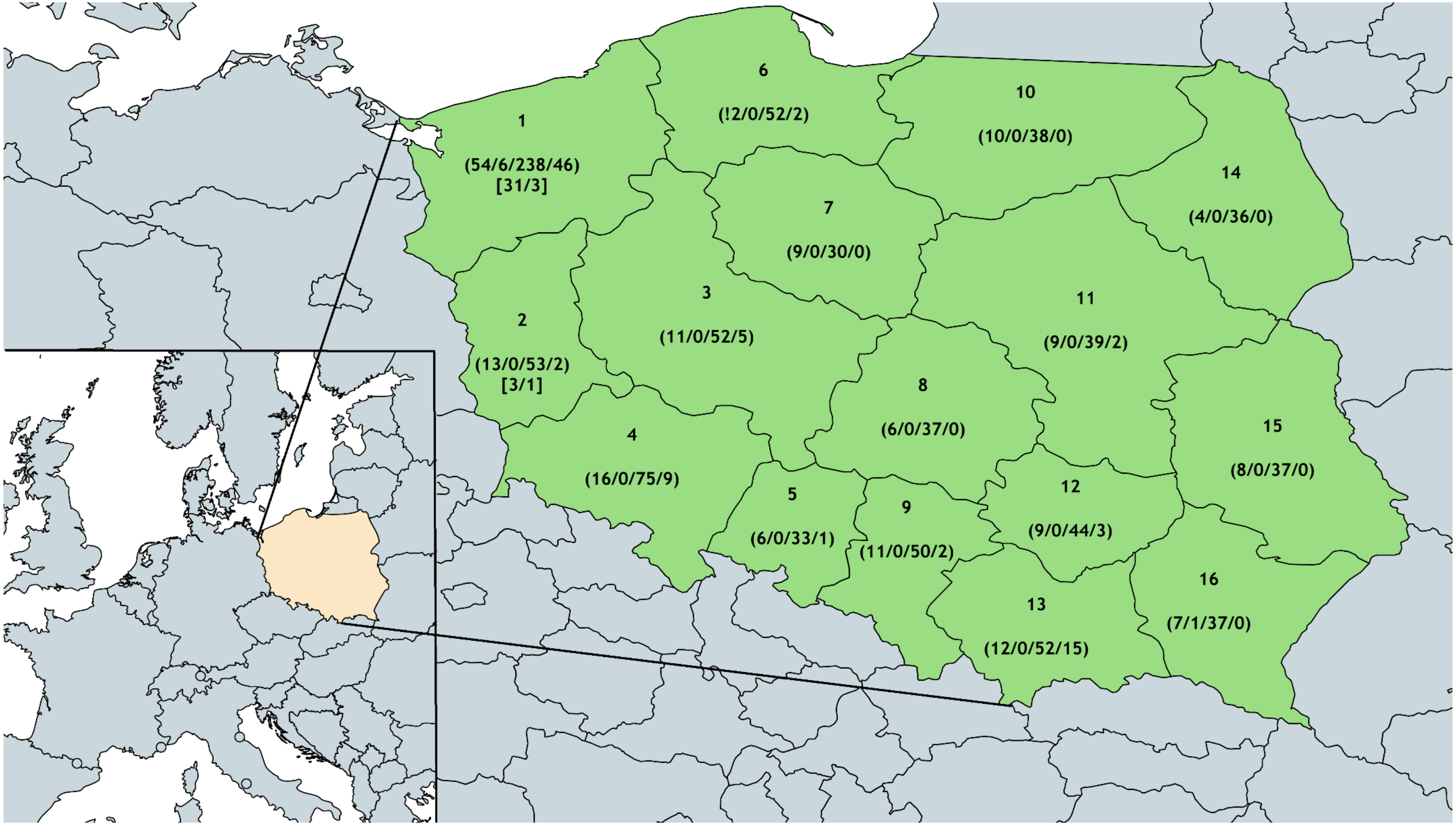

Figure S1. Number of ticks collected from humans in individual provinces in Poland in 2018–2022. The numbers in round brackets indicate the number of females/males/nymphs/larvae of Ixodes ricinus. The numbers in square brackets indicate the number of females/males of Dermacentor reticulatus. 1 – Zachodniopomorskie Province, 2 – Lubuskie Province, 3 – Wielkopolskie Province, 4 – Dolnośląskie Province, 5 Opolskie

Province, 6 – Pomorskie Province, 7 – Kujawsko-pomorskie Province, 8 – Łódzkie Province, 9 – Śląskie Province, 10 – Warmińsko-mazyrskie Province, 11 – Mazowieckie Province, 12 – Świętokrzyskie Province, 13 – Małopolskie Province, 14 – Podlaskie Province, 15 – Lubelskie Province, 16 – Podkarpackie Province.

Table S1. Prevalence of *Borreliaceae* species in ticks removed from humans in different provinces of Poland.

Province	Spirochete species (N/%)									Total (n/N/%)
	BA	BG	BB	BV	BS	BBI	BCL	BCR	BM	
Ixodes ricinus										
Zachodniopomorskie	30/41.1	3/4.1	10/13.7	5/6.8	9/12.3	6/8.2		1/1.4	9/12.3	344/73/21.2
Lubuskie	8/66.7		2/16.7						2/16.7	68/12/17.6
Wielkopolskie	6/60		1/10	2/20					1/10	68/10/14.7
Dolnośląskie	11/52.4	3/14.3		3/14.3	1/4.8				3/14.3	100/21/21
Opolskie	3/75								1/25	40/4/10
Pomorskie	8/50		5/31.3			2/12.5			1/6.8	66/16/24.2
Kujawsko-pomorskie	4/100									39/4/10.3
Łódzkie	3/60	1/20							1/20	43/5/11.6
Śląskie	5/33.3	1/6.7	2/13.3	2/13.3	2/13.3		1/6.7		2/13.3	63/15/23.8
Warmińsko-mazurskie	3/42.9		1/14.3			1/14.3			2/28.6	48/7/14.6
Mazowieckie	5/62.5		1/12.5		2/25					50/8/16
Świętokrzyskie	4/50	1/12.5	2/25		1/12.5					56/8/14.3
Małopolskie	1/9.1		4/36.4	1/9.1					5/45.5	79/11/13.9
Podlaskie	2/28.6		3/42.9	1/14.3					1/14.3	40/7/17.5
Lubelskie	4/50	2/25			1/12.5				1/12.5	45/8/17.8
Podkarpackie	3/30	1/10	2/20	2/20	1/10				1/10	45/10/22.2
Total	100/45.7	12/5.5	33/15.1	16/7.3	17/7.8	9/4.1	1/0.5	1/0.5	30/13.7	1194/219/18.3
Dermacentor reticulatus										
Zachodniopomorskie	5/41.7	1/8.3		1/8.3			1/8.3		4/33.3	34/12/35.3
Lubuskie									1/100	4/1/25
Total	5/38.5	1/7.7		1/7.7			1/7.7		5/38.5	38/13/34.2

N – number of infected; n – number of tested

BA – *Borreliella afzelii*, BG – *Bl. garinii*, BB – *Bl. burgdorferi*, BV – *Bl. valaisiana*, BS – *Bl. spielmanii*, BBI – *Bl. bissettiae*, BCL – *Bl. californiensis*, BCR – *Bl. carolinensis*, BM – *Borrelia miyamotoi*.

Table S2. *mag-trnI* intergenic spaces mean genetic distance within individual *Borreliaceae* species detected in host seeking ticks from Northern Poland.

Spirochete species	Mean genetic dinstance within species
Bl.afzelii	0.0037
Bl. garinii	0.0037
Bl.burgdorferi	0.0093
Bl.valaisiana	0.0069
Bl.spielmanii	0.007
Bl.bissettiae	0.0086
Bl.californiensis	0.0017
Bl.carolinensis	0.0045
B.miyamotoi	0.0021

Table S3. MEGA 11 results of mean distance between *Borreliaceae* species obtained on the basis of intergenic spacer (IGS) of 3-methyladenine glycosylase (*mag*) and tRNA-Ile (*trnI*) genes sequence fragment comparison.

	BG	BV	BA	BS	BB	BCL	BBI	BCR
Bl.valaisiana	0.1890							
Bl.afzelii	0.1638	0.2272						
Bl.spielmanii	0.1348	0.1682	0.1127					
Bl.burgdorferi	0.1995	0.2091	0.2401	0.2542				
Bl.californiensis	0.1410	0.1598	0.1974	0.1835	0.1315			
Bl.bissettiae	0.2283	0.2639	0.2726	0.2813	0.1452	0.1116		
Bl.carolinensis	0.1968	0.2405	0.2488	0.2605	0.1015	0.1030	0.0868	
B.miyamotoi	0.6566	0.6350	0.6910	0.7120	0.6512	0.5966	0.6422	0.6485

BG – *Borreliella garinii*, BV – *Bl. valaisiana*, BA – *Bl. afzelii*, BS – *Bl. spielmanii*, BB – *Bl. burgdorferi*, BCL – *Bl. californiensis*, BBI – *Bl. bissettiae*, BCR – *Bl. carolinensis.*

Table S4. *flaB* gene mean genetic distance within individual *Borreliaceae* species detected in host seeking ticks from Northern Poland.

Spirochete species	Mean genetic dinstance within species
Bl.afzelii	0.0009
Bl. garinii	0.0081
Bl.burgdorferi	0.0031
Bl.valaisiana	0.0013
Bl.spielmanii	0.0015
Bl.bissettiae	0.0023
Bl.californiensis	0.0017
Bl.carolinensis	0.0025
B.miyamotoi	0.0

Table S5. MEGA 11 results of mean distance between *Borreliaceae* species obtained on the basis of *flaB* gene sequence fragment comparison.

	BG	BV	BA	BS	BB	BCL	BBI	BCR
Bl.valaisiana	0.0466							
Bl.afzelii	0.0540	0.0451						
Bl.spielmanii	0.0526	0.0526	0.0463					
Bl.burgdorferi	0.0668	0.0577	0.0642	0.0635				
Bl.californiensis	0.0620	0.0520	0.0597	0.0607	0.0345			
Bl.bissettiae	0.0546	0.0497	0.0540	0.0580	0.0395	0.0296		
Bl.carolinensis	0.0550	0.0465	0.0508	0.0522	0.0384	0.0282	0.0067	
B.miyamotoi	0.1822	0.1693	0.1724	0.1775	0.1856	0.1829	0.1863	0.1804

BG – *Borreliella garinii*, BV – *Bl. valaisiana*, BA – *Bl. afzelii*, BS – *Bl. spielmanii*, BB – *Bl. burgdorferi*, BCL – *Bl. californiensis*, BBI – *Bl. bissettiae*, BCR – *Bl. carolinensis.*
